# Supplementary material for: An Economic Evaluation of the Adjuvanted Quadrivalent Influenza Vaccine Compared with Standard-Dose Quadrivalent Influenza Vaccine in the Spanish Older Adult Population
Source: Vaccines (Basel). 2022 Aug 20;10(8):1360. doi: 10.3390/vaccines10081360 (PMC9412909; doi:10.3390/vaccines10081360)
Supplement: Supplementary file 1 [file vaccines-10-01360-s001.zip › vaccines-1828956-supplementary.pdf]

Drummond checklist for the manuscript “An Economic Evaluation of the Adjuvanted Quadrivalent Influenza Vaccine Compared with Standard-Dose Quadrivalent Influenza Vaccine in the Spanish Older Adult Population” by A. Fochesato et al.

Item		Yes	No	Not clear	Not appropriate
**Study design**					
1.	The research question is stated.	☐x	☐	☐	
2.	The economic importance of the research question is stated.	☐x	☐	☐	
3.	The viewpoint(s) of the analysis are clearly stated and justified.	☐x	☐	☐	
4.	The rationale for choosing alternative programmes or interventions compared is stated.	☐x	☐	☐	
5.	The alternatives being compared are clearly described.	☐x	☐	☐	
6.	The form of economic evaluation used is stated.	☐x	☐	☐	
7.	The choice of form of economic evaluation is justified in relation to the questions addressed.	☐x	☐	☐	
**Data collection**					
8.	The source(s) of effectiveness estimates used are stated.	☐x	☐	☐	
9.	Details of the design and results of effectiveness study are given (if based on a single study).	☐	☐	☐	☐x
10.	Details of the methods of synthesis or meta-analysis of estimates are given (if based on a synthesis of a number of effectiveness studies).	☐	☐	☐	☐x
11.	The primary outcome measure(s) for the economic evaluation are clearly stated.	☐x	☐	☐	
12.	Methods to value benefits are stated.	☐x	☐	☐	☐
13.	Details of the subjects from whom valuations were obtained were given.	☐	☐	☐	☐x
14.	Productivity changes (if included) are reported separately.	☐	☐	☐	☐x
15.	The relevance of productivity changes to the study question is discussed.	☐	☐	☐	☐x
16.	Quantities of resource use are reported separately from their unit costs.	☐x	☐	☐	
17.	Methods for the estimation of quantities and unit costs are described.	☐x	☐	☐	
18.	Currency and price data are recorded.	☐x	☐	☐	
19.	Details of currency of price adjustments for inflation or currency conversion are given.	☐	☐	☐	x
20.	Details of any model used are given.	☐x	☐	☐	☐
21.	The choice of model used and the key parameters on which it is based are justified.	☐x	☐	☐	☐
**Analysis and interpretation of results**					
22.	Time horizon of costs and benefits is stated.	☐x	☐	☐	☐
23.	The discount rate(s) is stated.	☐x	☐	☐	☐
24.	The choice of discount rate(s) is justified.	☐x	☐	☐	☐
25.	An explanation is given if costs and benefits are not discounted.	☐	☐	☐	☐x
26.	Details of statistical tests and confidence intervals are given for stochastic data.	☐	☐	☐	☐x

27.	The approach to sensitivity analysis is given.	☐x	☐	☐	☐
28.	The choice of variables for sensitivity analysis is justified.	☐x	☐	☐	☐
29.	The ranges over which the variables are varied are justified.	☐x	☐	☐	☐
30.	Relevant alternatives are compared.	☐x	☐	☐	☐
31.	Incremental analysis is reported.	☐	☐	☐	☐x
32.	Major outcomes are presented in a disaggregated as well as aggregated form.	☐x	☐	☐	
33.	The answer to the study question is given.	☐x	☐	☐	
34.	Conclusions follow from the data reported.	☐x	☐	☐	
35.	Conclusions are accompanied by the appropriate caveats.	☐x	☐	☐	
